# Comparing the effects of Al-based coagulants in waste activated sludge anaerobic digestion: Methane yield, kinetics and sludge implications

**DOI:** 10.1016/j.heliyon.2024.e29282

**Published:** 2024-04-07

**Authors:** Francesco Pasciucco, Erika Pasciucco, Alessio Castagnoli, Renato Iannelli, Isabella Pecorini

**Affiliations:** Department of Energy, Systems, Territory and Construction Engineering (DESTEC), University of Pisa, 56122, Pisa, Italy

**Keywords:** Anaerobic digestion, Waste activated sludge, Methane production, Poly aluminum chloride, Aluminum sulfate, Coagulants

## Abstract

Due to its effectiveness and ease of application, the process of flocculation and coagulation is often used for pollution removal in wastewater treatment. Most of these coagulants precipitate and accumulate in waste activated sludge (WAS), and could negatively affect sludge treatments, as observed for anaerobic digestion. Nowadays, wastewater treatment plants (WWTPs) are widely discussed because of the current paradigm shift from linear to circular economy, and the treatments performed at the facility should be planned to avoid or reduce adverse effects on other processes. The aim of this study was to compare the impact of poly aluminum chloride (PAC) and aluminum sulfate (AS) on WAS anaerobic digestion, by feeding replicate serum reactors with different levels of coagulant (5, 10 and 20 mg Al/g TS). Reactors without the addition of any coagulants represented the control group. Results revealed that Al-based coagulants inhibited methane production, which decreased as the coagulant addition increased. The inhibition was much more severe in AS-conditioned reactors, showing average reductions in methane yield from 14.4 to 31.7%, compared to the control (167.76 ± 1.88 mL CH_4_/g VS). Analytical analysis, FTIR and SEM investigations revealed that the addition of coagulants affected the initial conditions of the anaerobic reactors, penalizing the solubilization, hydrolysis and acidogenesis phases. Furthermore, the massive formation of H_2_S in AS-conditioned reactors played a key role in the suppression of methane phase. On the other hand, the use of coagulant can promote the accumulation and recovery of nutrient in WAS, especially in terms of phosphorus. Our findings will expand research knowledge in this field and guide stakeholders in the choice of coagulants at full scale plant. Future research should focus on reducing the effect of coagulants on methane production by modifying or testing new types of flocculants.

## Highlights

• The impact of Al-based coagulants on sludge anaerobic digestion was evaluated.
• Solubilization, hydrolysis, acidogenesis and methane phases were suppressed.
• Poly aluminum chloride (PAC) inhibited methane production less severely.
• Aluminum sulfate (AS) and PAC showed opposite effects on H_2_S production.
• AS methane inhibition was increased by enriched sulfate-reducing bacteria.

## 1 Introduction

Nowadays, wastewater treatment plants (WWTPs) are widely discussed because of the current paradigm shift from linear to circular economy that they are facing [1].

Indeed, WWTPs are intended to embrace the biorefinery concept in the near future [2], promoting the sustainability of processes from infrastructure planning [3] to recovery and reuse of by-products [4].

In this context, WWTPs must satisfy the dual objective of protecting human health and mitigating the consumption of non-renewable resources [5]. Compliance with national and international water quality standards occurs through a treatment chain consisting of processes that may conflict with each other; therefore, the treatments performed at the facility should be planned to avoid or reduce adverse effects on other processes [6].

Due to its effectiveness and ease of application, the process of flocculation and coagulation is often used for pollution removal in wastewater treatment, including emerging contaminants [7] and nutrients [8]. The aim of the process is to destabilize the colloidal components and induce the small particles to clump together into bigger settleable flocs [9], by means of two steps: i) rapid mixing of coagulants via vigorous agitation and ii) flocculation to aggregate small particles into distinct flocs via soft agitation [10]. The operating times for rapid mixing and soft agitation phases are established based on previous tests, and generally range from 1 to 10 min and from 10 to 30 min, respectively [11]. Aluminum (Al) and iron (Fe)-based coagulants are widely applied because of their recognized efficiency, accessibility and affordability [12]: poly ferric sulfate (PFS), ferric chloride (FC), poly aluminum chloride (PAC) and aluminum sulfate (AS) are commonly used coagulants [13], with typical dosages ranging from 10 to 60 mg/L, depending on wastewater characteristics [14].

On the other hand, most of these coagulants precipitate and accumulate in waste activated sludge (WAS). According to the literature, Al concentration in WAS ranged from 2.6 mg/g [15] to 28.8 mg/g [16], while Fe concentration ranged from 1.9 to 15.4 mg/g [17], and could negatively affect treatments implemented for sludge stabilization, as reported in the case of anaerobic digestion. The process of anaerobic digestion of WAS is one of the most widespread large-scale technologies because of the biogas produced during the process [18], which allows to retrieve heat, electricity and methane, and offers the most effective energy input/output ratio compared to other biological and thermochemical conversion processes [19].

In the last few years, this topic has attracted great interest in the literature. Liu et al. [14] explored the influence of PFS on anaerobic digestion of WAS. Zhan et al. [20] studied the roles of FC. Wu et al. [21] compared the effects of PAC and PFS. The above studies focused on the underlying mechanisms of how coagulants affect methane yield during WAS anaerobic digestion; among them, only Zhan et al. [20] reported an increase in methane production from WAS samples conditioned by the addition of coagulants. Recently, Cainglet et al. [22] investigated the effects of inorganic coagulants and organic coagulants on the biological stabilization of sewage sludge, revealing that organic coagulants led to increased biogas production while reducing biomass biodegradability due to the presence of complex organic compounds. In general, Al-based coagulants are known to have negative impacts on methane production in WAS anaerobic digestion, especially affecting hydrolysis and acidogenesis [23]. In view of that, Chen et al. [24] observed that increasing the level of PAC up to 40 mg Al/g total suspended solids (TSS) decreased short-chain fatty acids (SCFAs) production by 35%.

Despite the latest developments, in authors’ knowledge, none of the previous studies compared the performance of PAC and AS on methane production. Liu et al. [25] investigated the presence of PAC and AS on the formation of aerobic granules; however, their effects on anaerobic digestion have not yet been addressed. The aim of this study was to compare the impact of PAC and AS in WAS anaerobic digestion, focusing on methane yield and sludge characteristics after coagulant addition.

The results obtained will expand research knowledge in this field and guide stakeholders in the choice of coagulants at full scale plant in the future, so as to evaluate the best compromise between pollution removal and resource recovery [26].

## 2 Materials and methods

### 2.1 Properties of WAS, inoculum sludge and Al-based coagulants

In most of the previous studies in the literature [14,20,21], WAS was retrieved from secondary clarifiers of municipal WWTPs, and then concentrated by gravity thickening in the laboratory. In our case study, the WAS was collected from a sludge thickening tank of a medium-size municipal WWTP in Tuscany, Italy. The thickening tank is placed downstream of the secondary clarifier tank and upstream of the anaerobic digestor, in order to take into account a situation as close as possible to real conditions. In the considered WWTP, no coagulants are dosed for wastewater treatment.

The inoculum sludge was collected from a long-term continuous WAS anaerobic digester operating under mesophilic conditions. Both WAS and inoculum sludge were stored at 4 °C for further analysis, and their main features are shown in Table 1. The main characteristic of the Al-based coagulants used in this study are reported in Table 2. PAC was obtained from C.M. Chimica Company (Pistoia, Italy), while AS was purchased from VWR Company (Milan, Italy).

**Table 1:** Main features of waste activated sludge (WAS) and inoculum sludge.

Parameter	Units	WAS	Inoculum sludge
pH		6.6 ± 0.2	7.8 ± 0.3
Total solids (TS)	% w/w	1.77 ± 0.003	0.65 ± 0.0002
Volatile solids (VS)	% TS	81.2 ± 0.13	56.01 ± 0.23
Total chemical oxygen demand (TCOD)	mg/L	26,500 ± 1,800	1,420 ± 170
Soluble total organic carbon (STOC)	mg/L	2,240 ± 120	644 ± 97
Soluble proteins	mg/L	194.7 ± 8.7	45.5 ± 4.2
Soluble carbohydrates	mg/L	88.5 ± 6.1	19.5 + 3.2
Soluble lipids	mg/L	3.5 ± 0.2	0.7 ± 0.03
Soluble ammonium	mg/L	252 ± 22	830 ± 120
Soluble phosphorus	mg/L	384 ± 18	142 ± 21

**Table 2:** Main features of poly aluminum chloride (PAC) and aluminum sulfate (AS).

Parameter	Units	PAC	AS
Assay (as Al_2_O_3_)	%	16–18	16.5–17.5
Heavy metals	ppm	≤10	≤20
Chloride (Cl)	ppm	≤220,000	≤100
Iron (Fe)	ppm	≤50	≤100
Not precipitated by NH_4_OH (as SO_4_)	%	–	≤0.3

### 2.2 Effect of different Al-based coagulant levels on WAS anaerobic digestion

To investigate the effect of PAC and AS on WAS anaerobic digestion, batch experiments were performed in replicate serum reactors (1000 mL of total volume, 600 mL of working volume). Each reactor was analyzed in triplicate, and the ratio of inoculum to substrate was determined based on VS content [27], following the standardized procedure developed by Angelidaki et al. [28] and adapted by Pecorini et al. [29].

First, each reactor was fed with 800 mL of WAS [14]. Then, the reactors were fed with different volumes of PAC and AS, according to the dosages and nomenclature indicated in Table 3, and stirred for 2 min at 120 rpm and 10 min at 60 rpm [30]. At the end of coagulant conditioning, samples of sludge were withdrawn with a syringe to evaluate the effect of coagulants on WAS in this intermediate phase.

**Table 3:** Nomenclature and dosages in replicate reactors of poly aluminum chloride (PAC) and aluminum sulfate (AS).

Reactor groups	Dosage of PAC (mg Al/g TS)	Dosage of AS (mg Al/g TS)
Control	0	0
PAC 5	5	0
PAC 10	10	0
PAC 20	20	0
AS 5	0	5
AS 10	0	10
AS 20	0	20

Finally, each reactor was fed with 250 mL of inoculum sludge and the pH was adjusted to 7 ± 0.1 using NaOH or HCl [14]. The reactors were flushed with inert gas (nitrogen) to guarantee anaerobic conditions in the headspace of the bottles, sealed tightly with a cap equipped with a ball valve to allow gas sampling and incubated at 37 ± 0.1 °C in a water bath. Daily, the reactors were shaken to ensure homogeneous conditions, and the methane and H_2_S content of biogas was monitored [31].

### 2.3 Kinetic analysis

For better interpretation of the kinetics of methane potential (BMP) tests, experimental data of cumulative methane production curves were fitted by the Modified Gompertz model (Eq. (1)) [14].

(1) $$\text{BMP}\;\left(t\right)=A\times \exp\left\{-\exp\left[\frac{\mu_{m}\times e}{A}\;\left(\lambda -t\right)+1\right]\right\}$$

where:

BMP(t) = cumulative methane production at time t;

A = maximum methane production potential (mL/g VS);

μ_m_ = maximum methane production rate (mL/g VS · d);

λ = lag-phase duration (d);

t = time (d);

e = exp(1).

Kinetic parameters of Equation (1) were calculated by means of least-square linear regression using a calculation software [32]. The correlation between Al-based coagulant addition with the maximum methane production potential (A), maximum methane production rate (μ_m_) and lag-phase duration (λ) was simulated by exponential equation [14].

### 2.4 Effect of Al-based coagulants on sludge structure and composition

As mentioned, samples of sludge were withdrawn after coagulant conditioning for further analysis. In addition to the variation in the contents of TS, VS and the main soluble components, the interaction between PAC, AS and complex organic mixture was explored using the Fourier Transform Infrared Spectroscopy (FTIR) [33] and Scanning Electron Microscopy (SEM) [34].

### 2.5 Analytical methods

The values of TCOD, STOC, soluble proteins, soluble carbohydrates, soluble lipids, soluble ammonium and soluble total phosphorus were performed according to the Standard Methods [35]. To obtain soluble parameters, sludge samples were centrifuged and filtered using 0.45 μm disposable filters before being analyzed [36].

Gravimetric measurements were made for TS and VS. The sludge samples were first dried for 24 h at 105 °C, and then burned for 4 h at 550 °C. The pH was measured using a pH meter PC 700 by Euthech Instruments.

Concerning biogas production, a pressure gauge (Model HD2304.0, Delta Ohm S.r.L., Italy) was used to monitor the headspace pressure. The biogas volume was then computed by converting the headspace volume to standard conditions of temperature and pressure (273.15 K and 1 atm, respectively) [31]. The methane and H_2_S contents were analyzed by collecting biogas in Tedlar Sample Bags and using a Gas Chromatography system (INFICON 3000 Micro GC).

SEM analysis was conducted using FEI Quanta 450 FEG operated at 15 kV. Spectrum GX spectroscope (PerkinElmer) was used for collecting FTIR spectra in the range of 4000 to 400 cm-1. In this case, the KBr pellet method was used, where 5 mg of sample was mixed with KBr in a 1:5 ratio.

### 2.6 Statistical analysis

The experiment was conducted in triplicate, and statistical significance was identified at p < 0.05 [37], implementing an analysis of variance with least significant difference test [14]. The findings were presented in the form of mean ± standard deviation.

## 3 Results and discussion

### 3.1 Impact of PAC and AS on methane yield and kinetics

**Fig. 1:**
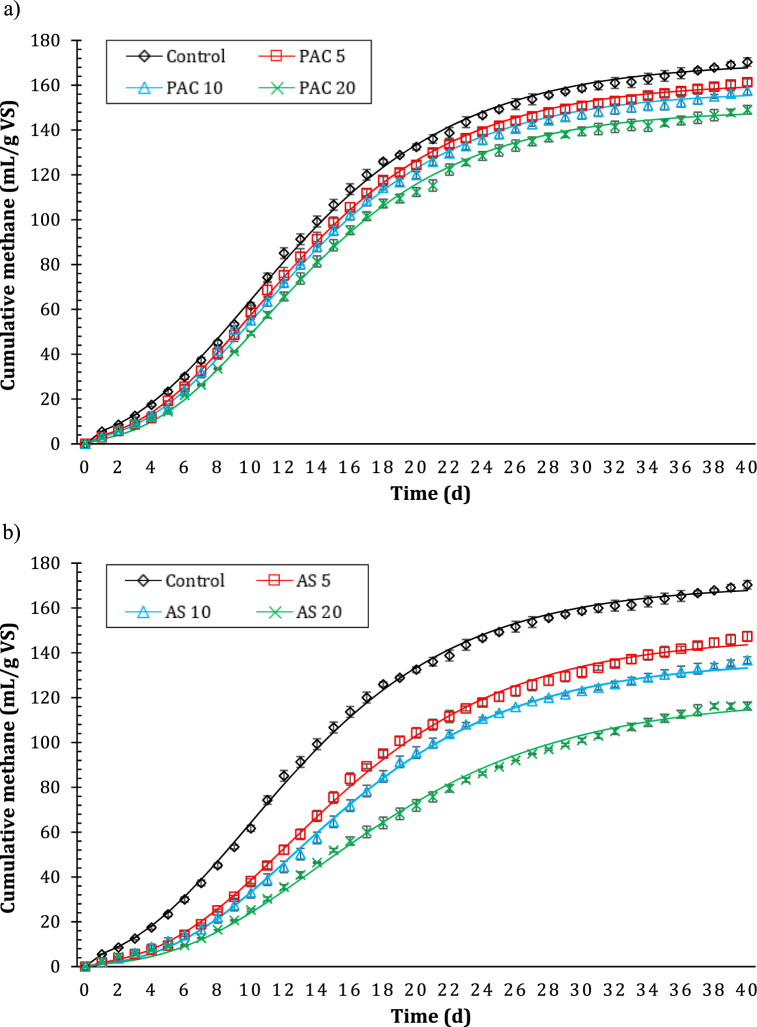
Cumulative methane production in control reactor (no coagulant addition) compared with poly aluminum chloride (PAC)-conditioned reactors (a) and aluminum sulfate (AS)-conditioned reactors (b). The numbers associated with the reactors indicate the coagulant level in terms of mg Al/g TS.

The cumulative curves of methane production from WAS anaerobic digestion were reported in Fig. 1, considering different levels of PAC and AS coagulants.

Specifically, in Fig. 1a, the cumulative production of methane in control and PAC-conditioned reactors were compared; whereas, AS-conditioned reactors were compared to the control in Fig. 1b. BMP curves of control, PAC and AS group reactors were simulated according to the Modified Gompertz model (R^2^ > 0.9827 in all cases, indicating that the fit between experimental data and model was satisfying) [38] and were shown in two different charts for better visualization.

Statistically, the experimental test was significant, as methane production in the reactor increased with the digestion time from 0 to 40 days, and no significant increases were observed after 40 days (p > 0.05), which means that the anaerobic digestion process was substantially completed [14].

As shown in Fig. 1, the BMP curves of control, PAC-conditioned (Fig. 1a) and AS-conditioned (Fig. 1b) reactors had similar trends, but the cumulative methane production was inhibited in reactors affected by Al-coagulant addition.

In particular, the maximum methane yield was reached in control reactor, where no coagulants were added for WAS anaerobic digestion, and was equal to 167.76 ± 1.88 mL CH_4_/g VS. On the other hand, as mentioned, the addition of PAC and AS led to a decrease in methane production, and the maximum methane yield decreased as the level of coagulant in the reactors increased from 0 to 20 mg Al/g TS.

The reactors fed with AS showed a more severe inhibition of the anaerobic digestion process, as the maximum methane productions of AS-conditioned reactors were always lower compared to those measured in the reactors of the PAC group. AS 5, AS 10 and AS 20 reactors generated a potential methane production of 143.58 ± 2.19 mL CH_4_/g VS, 133.24 ± 1.54 mL CH_4_/g VS and 114.54 ± 1.79 mL CH_4_/g VS, respectively. Conversely, maximum methane yields in PAC 5 and PAC 10 reactors were quite close to those of the control reactor, showing average reductions of 5.1% (159.13 ± 1.51 mL CH_4_/g VS) and 7.4% (155.41 ± 1.77 mL CH_4_/g VS), respectively. The addition of 20 mg Al/g TS of PAC generated a more evident reduction in methane yield, accounting for 12.3% on average (147.12 ± 1.95 mL CH_4_/g VS); however, it was still a greater production than that associated with AS group reactors. In this regard, Wu et al. [21] explored additional PAC gradients (up to 30 mg Al/g TS), confirming the increase in methane inhibition.

The above findings were consistent with Liu et al. [14] and Wu et al. [21]; according to the authors, the presence of Fe and Al-based coagulants inhibited the production of methane, showing maximum methane yields in control reactors similar to ours.

The kinetic parameters of the BMP tests were computed according to the Modified Gompertz model, in order to provide an in-depth investigation of the effects of Al-based coagulants on WAS anaerobic digestion.

**Fig. 2:**
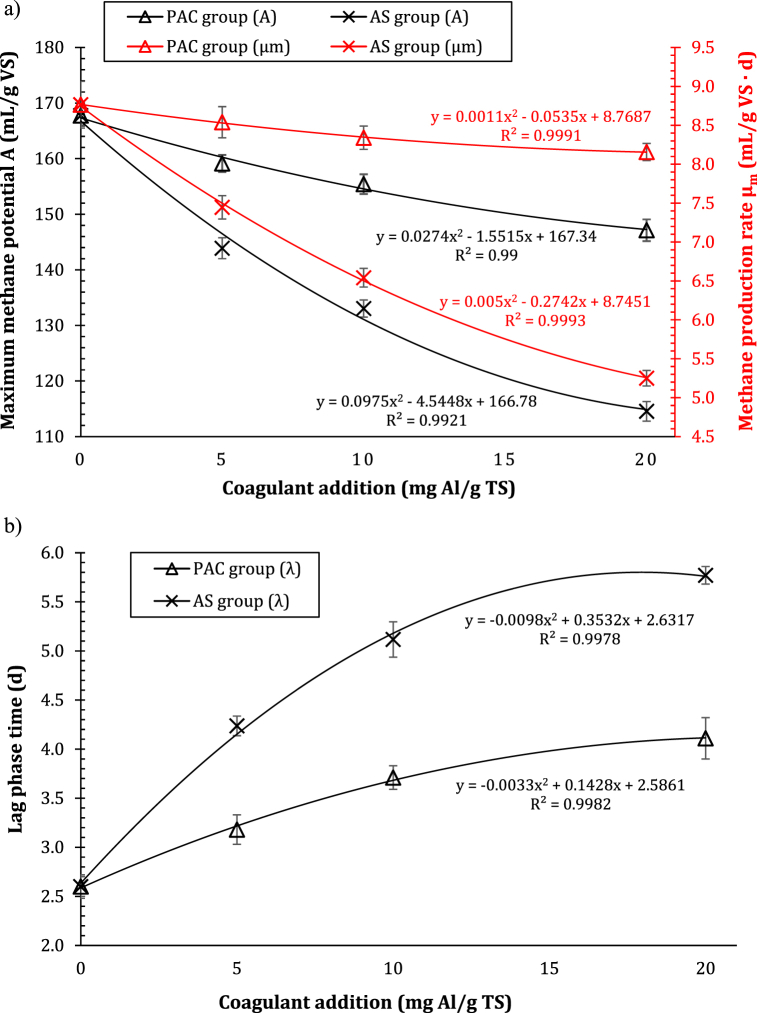
Maximum methane production potential (a, in black), maximum methane production rate (a, in red) and lag-phase duration (b), depending on different levels of poly aluminum chloride (PAC) and aluminum sulfate (AS).

In Fig. 2a, the maximum methane production potential (A, mL/g VS) and maximum methane production rate (μ_m_, mL/g VS d) were shown, depending on PAC and AS addition. The negative relationship existing between the maximum methane production potential and the amount of dosed Al-based coagulant was more noticeable for the reactors of the AS group than for those of the PAC group, and was displayed in black in Fig. 2a.

At the same time, the maximum methane production rate (μ_m_) decreased as the coagulant level increased (in red in Fig. 2a). Anyway, it should be noted that the reduction in μ_m_ values was much lower in the reactors of PAC group (from 2.6 to 6.9%, on average) than the reactors of AS group (from 15.0 to 40.1%, on average), suggesting that AS had a greater impact on methanogenic activity than PAC [14].

On the contrary, the lag-phase duration (λ, d) showed an opposite trend, as it increased with the increasing coagulant level, as shown in Fig. 2b. The highest value of λ in PAC-conditioned reactors was 1.58 times than that in control reactor (2.6 days), while the maximum lag-phase duration in AS-conditioned reactors was 2.22 times higher. Again, the lag-phase duration in all AS-conditioned reactors was higher than those computed for PAC group reactors, indicating that AS extended the start-up period of the reactors more than PAC [14].

### 3.2 Inhibitory factors in methane production by Al-based coagulants

#### 3.2.1 Insights about solubilization and hydrolysis phases

The process of anaerobic digestion consists of various sequential steps: solubilization of particulate organic matter, hydrolysis of organic macromolecules (e.g., proteins, carbohydrates) into monomers, acidogenesis of organic micromolecules (e.g., amino acids), acetogenesis of acid fermentation products and methanogenesis of hydrogen and acetates [39].

The results discussed in previous section highlighted that the potential methane production was negatively affected by the addition of Al-based coagulants. Possible reasons for this phenomenon can be deduced from previous studies in the literature, which have already analyzed in-depth the contribution of coagulants in each phase of WAS anaerobic digestion, and proved to be consistent with our findings.

According to the literature, solubilization represents a crucial rate-limiting step, and can be evaluated based on the release of soluble proteins and carbohydrates. Liu et al. [14] and Wu et al. [21] assessed the degree of solubilization after 3 days of anaerobic digestion and thermal pretreatment of WAS, respectively. In this study, we focused on the concentration of soluble proteins, carbohydrates and lipids in WAS after the addition of PAC and AS, in order to consider the immediate effect of Al-based coagulants and the starting condition of the anaerobic digestion process in reactors.

**Fig. 3:**
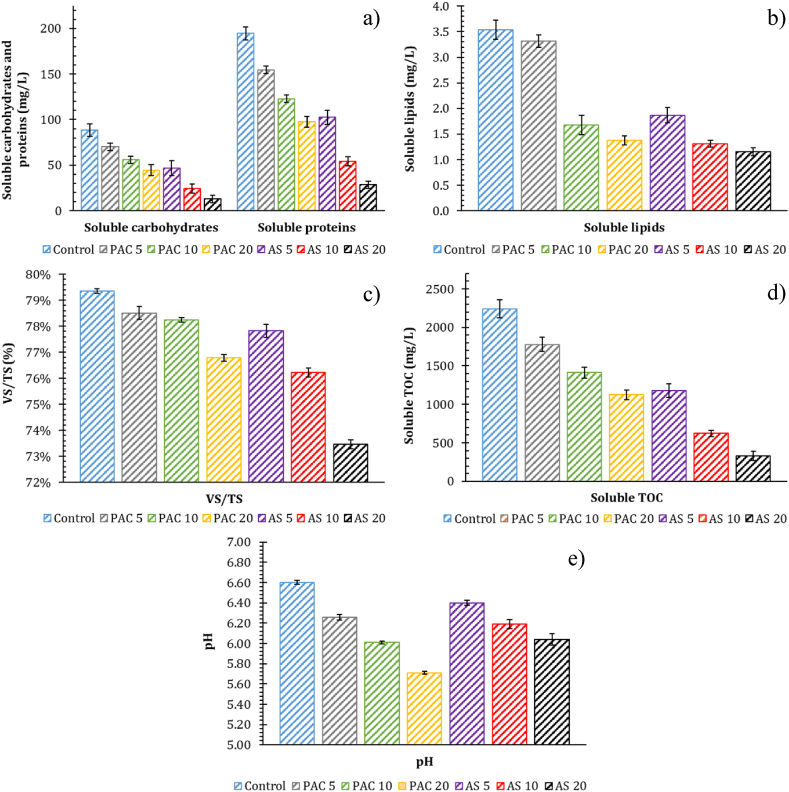
Values of soluble carbohydrates and proteins (a), soluble lipids (b), VS/TS (c), soluble TOC (d) and pH (e) in waste activated sludge (WAS) after the addition of different poly aluminum chloride (PAC) and aluminum sulfate (AS) levels. The associated numbers in the legend indicate the coagulant level in terms of mg Al/g TS. No coagulants were dosed in the control.

As reported in Fig. 3, the concentration of soluble carbohydrates (Fig. 3a), soluble proteins (Fig. 3a) and soluble lipids (Fig. 3b) decrease as the level of Al-based coagulants increased, highlighting that the reactors of PAC and AS groups contained a smaller amount of readily biodegradable organic compounds compared to the control reactor. In particular, coagulation action was more evident in the reactors of AS group, showing average reductions ranging from 47 to 85% in soluble carbohydrates and proteins, and from 43.5% to 67.4% in soluble lipids.

Also, VS content and size of sludge flocs are parameters closely associated with the solubilization properties of WAS [40].

Concerning VS content in WAS, the ratio of VS to TS in control, PAC-conditioned and AS-conditioned reactors after coagulant addition was reported in Fig. 3c. As can be seen, the percentage of volatile compounds decreased with increasing level of Al-based coagulants. Higher reductions were noted in AS-conditioned reactors, where the ratio VS/TS ranged from 77.84 ± 0.25% in AS 5 to 73.46 ± 0.18% in AS 20, compared to 79.36 ± 0.08% of the control reactor. Consistently with previous data, reductions of VS/TS in PAC group reactors were significant with the addition of 20 mg Al/g TS.

Regarding WAS floc size, it is widely recognized that sludge solubilization and hydrolysis occur more rapidly with smaller particle size, due to the higher surface-to-volume ratio [24]. As reported by previous studies, it can be stated coagulant effectiveness on COD and TOC removal results in enhanced floc aggregation and larger particle size [21]. The concentration of soluble TOC after coagulant addition was shown in Fig. 3d. In each group, soluble TOC decreased as the level of coagulant increased, with a maximum average reduction equal to 80.4% in AS 20 compared to the control reactor. Considering the same level of Al-based coagulant dosed (mg Al/g TS), the concentration of soluble TOC was always lower in AS-conditioned reactors; therefore, it is reasonable to assume that sludge particle size was as follows: AS > PAC > control.

#### 3.2.2 Assumptions about acidogenesis phase

Specific measurements on acidogenesis phase were not investigated in this study; however, some assumptions can be deduced from previous works.

The acidogenesis phase is a basic step in the anaerobic digestion process. Indeed, by-products of solubilization and hydrolysis undergo processes for the production of short-chain fatty acids (SCFA) during this phase, which will then be converted into methane.

Given the above, Chen et al. [24] demonstrated that the addition of PAC reduced the pH of WAS. In general, Al-based coagulants should generate this phenomenon, probably due to the presence of aluminum ions that can promote the polarization of water molecules and cause the loss of protons [41].

In accordance with that, the pH value decreased in reactors after the addition of Al-based coagulant, and decreased with increasing the coagulant level in each group (Fig. 3e). Some studies reported that lower the pH value the higher production of SCFA in the range of 5–7 [42]; however, Chen et al. [24] showed that the production of SCFA decreased as the volume of PAC increased, due to inhibitory effects in solubilization and hydrolysis processes, thus affecting the following steps of acetate degradation and methane production.

Similar outcomes were found by Kim & Jung [43], who demonstrated that the addition of Al-based coagulants suppressed the production of SCFA, confirming that the acidogenesis phase is another limiting-step of the whole process.

Anyway, it should be noted that other studies in the literature reported an increase in SCFAs production at neutral pH, as it is beneficial to microorganisms related to acidification [44].

#### 3.2.3 Insights about the formation of H_2_S

The above discussions explained how the addition of Al-based coagulants affected and inhibited the production of methane, going through experimental data and findings from the literature. Indeed, the content of soluble organic matter, the size of the flocs and the pH at the beginning of the anaerobic process play fundamental roles.

Based on this, it is worth noting that the amount of soluble organic matter in the AS 5 reactor was higher than that in PAC 20 reactor (Fig. 3). At the same time, the pH values in AS group reactors were generally higher than PAC group reactors. However, there is another factor to take into account to explain these apparent contradictions, and assume why the yield of methane was always lower in AS group reactors.

Both organic and inorganic sulfur can be reduced to H_2_S during anaerobic digestion, simultaneously with the production of biogas from organic carbon [45].

**Fig. 4:**
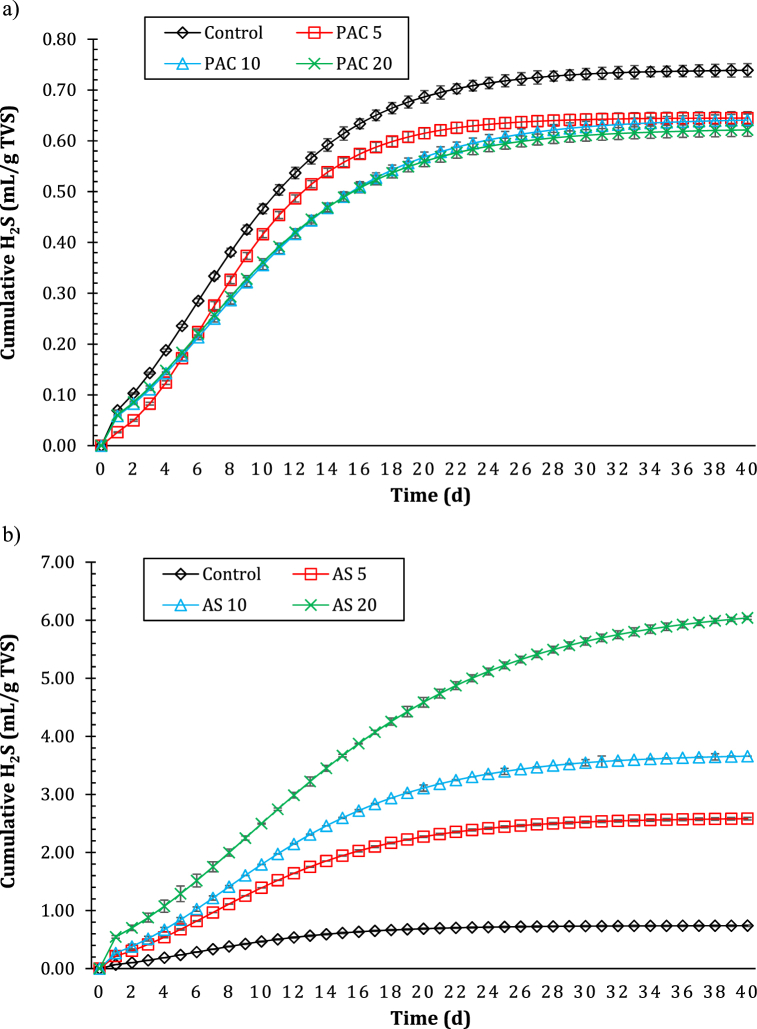
Cumulative H_2_S production in control reactor (no coagulant addition) compared with poly aluminum chloride (PAC)-conditioned reactors (a) and aluminum sulfate (AS)-conditioned reactors (b). The numbers associated with the reactors indicate the coagulant level in terms of mg Al/g TS.

The formation of H_2_S in each reactor was reported in Fig. 4. The production of H_2_S in PAC group reactors (from 6.22E-01 ± 0.0118 to 6.45E-01 ± 0.0125 mL H_2_S/g VS, Fig. 4a) was slightly lower than the control reactor (7.39E-01 ± 0.013 mL H_2_S/g VS); on the other hand, the H_2_S produced in AS group reactors was one order of magnitude greater, and increased as the level of AS coagulant increased (from 2.58 ± 0.022 to 6.04 ± 0.026 mL H_2_S/g VS, Fig. 4b).

This was an expected result, due to the presence of sulfate in AS, and similar trends were found in Wu et al. [21], who compared PAC and PFS. As reported by the authors, sulfate-reducing bacteria play a key role and are competitors of methanogenic bacteria, as they can use hydrogen and organic compounds (e.g. acetic acid) to produce H_2_S [46].

Indeed, sulfate-reducing bacteria reduce SO_4_^2−^ into H_2_S. The reduction of SO_4_^2−^ by both H_2_ and acetic acid as electron donors are promoted from a thermodynamic point of view then methanogenesis of H_2_ and acetic acid (Eqs. (1)–(4)) [45].

SO_4_^2−^ + 4H_2_

<svg xmlns="http://www.w3.org/2000/svg" version="1.0" width="20.666667pt" height="16.000000pt" viewBox="0 0 20.666667 16.000000" preserveAspectRatio="xMidYMid meet"><metadata>
Created by potrace 1.16, written by Peter Selinger 2001-2019
</metadata><g transform="translate(1.000000,15.000000) scale(0.019444,-0.019444)" fill="currentColor" stroke="none"><path d="M0 440 l0 -40 480 0 480 0 0 40 0 40 -480 0 -480 0 0 -40z M0 280 l0 -40 480 0 480 0 0 40 0 40 -480 0 -480 0 0 -40z"/></g></svg>

H_2_S + 4H_2_O + 2OH^−^ (1)

(2) CO_2_ + 4H_2_CH_4_ + 2H_2_O

(3) SO_4_^2−^ + CH_3_COOHH_2_S + 2HCO_3_^−^

(4) CH_3_COOHCH_4_ + CO_2_

The large amount of sulfate in AS enriched the sulfate-reducing biomass in AS group reactors, as shown by the cumulative H_2_S formation curves in Fig. 4b; therefore, it can be stated that the inhibition of methane yield was much severe by AS due to the development of sulfate-reducing bacteria in AS-conditioned reactors, as suggested by Wu et al. [21].

### 3.3 Structure of WAS after Al-based coagulant conditioning

#### 3.3.1 FTIR analysis

**Fig. 5:**
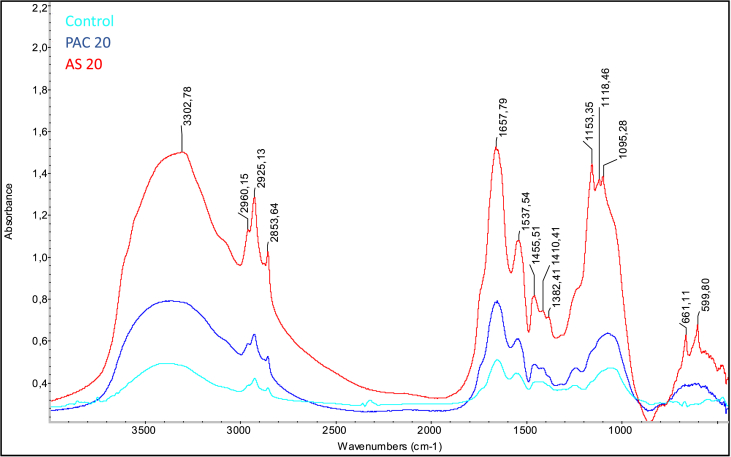
FTIR analysis for control (no coagulant addition), PAC 20 (20 mg Al/g TS of poly aluminum chloride) and AS 20 (20 mg Al/g TS of aluminum sulfate) samples.

The influence of PAC and AS coagulants in sludge structure, in terms of functional groups, was investigated by FTIR spectroscopy, and the corresponding IR bands were assigned based on the existing literature. In Fig. 5, the spectra of control, PAC 20 and AS 20 samples are compared, since the differences are more visible; in supplementary material, the individual spectra are shown in detail.

The spectrum showed a rather wide band around 3300 cm^−1^, which corresponded both to the O–H stretching of the hydroxyl group and the N–H stretching of the secondary amides [21].

The addition of 20 mg Al/g TS of PAC and AS resulted in a band shift at lower vibrational frequencies, from 3393 cm^−1^ (control reactor) to 3356 and 3302 cm^−1^, respectively. The shift suggested that Al-based coagulants showed significant differences when incorporating molecules containing O–H and N–H groups [25].

The peaks in the region of the spectrum between 3000 and 2850 cm^−1^ were associated with the symmetric (2853 cm^−1^) and asymmetric (2925 cm^−1^) C–H stretching of the methylene groups. The existence of proteins in all the investigated samples was confirmed by the presence of two characteristic bands in the spectrum: the band around 1657 cm^−1^, which was referred to the C=O stretching of amide I, and the band around 1537 cm^−1^, which was associated with the N–H and C–N bond stretching of amide II [47]. However, in this case only a weak shift was observed for the bands corresponding to the vibrational N–H stretching of amide II.

The region of the spectrum at frequencies around 1000 cm^−1^ was characteristic of polysaccharides. Compared to the control (band at 1049 cm^−1^), the bands of PAC 20 and AS 20 were shifted to higher vibrational frequencies. The peaks appeared more defined after the addition of 20 mg Al/g TS of AS (bands around 1153-1095 cm^−1^), and can be referred with the C–O–C vibrational stretching of polysaccharides. This resulted in the strong interaction of aluminum sulfate toward polysaccharides, which was highlighted by the shift of the peaks to higher vibrational frequencies.

Furthermore, the spectral pattern at vibrational frequencies <950 cm^−1^ (fingerprint region) emphasized the role of AS 20 as a coagulant. In fact, the presence of two well defined peaks around 600 cm^−1^ was observed only after the addition of 20 mg Al/g TS of AS; whereas, both in the case of control and PAC 20 samples, this region was characterized by broad bands with weakly marked peaks. The presence of these bands confirmed the existence of groups such as C–O, C–O–C and P=O in polyesters and polysaccharides, and demonstrated the superiority of AS as a coagulant also towards phosphate and sulfate groups [25,27].

#### 3.3.2 SEM analysis

To further understand the changes in the morphology of samples after the addition of PAC and AS, SEM images were collected at different magnitude range and concentration of coagulant.

**Fig. 6:**
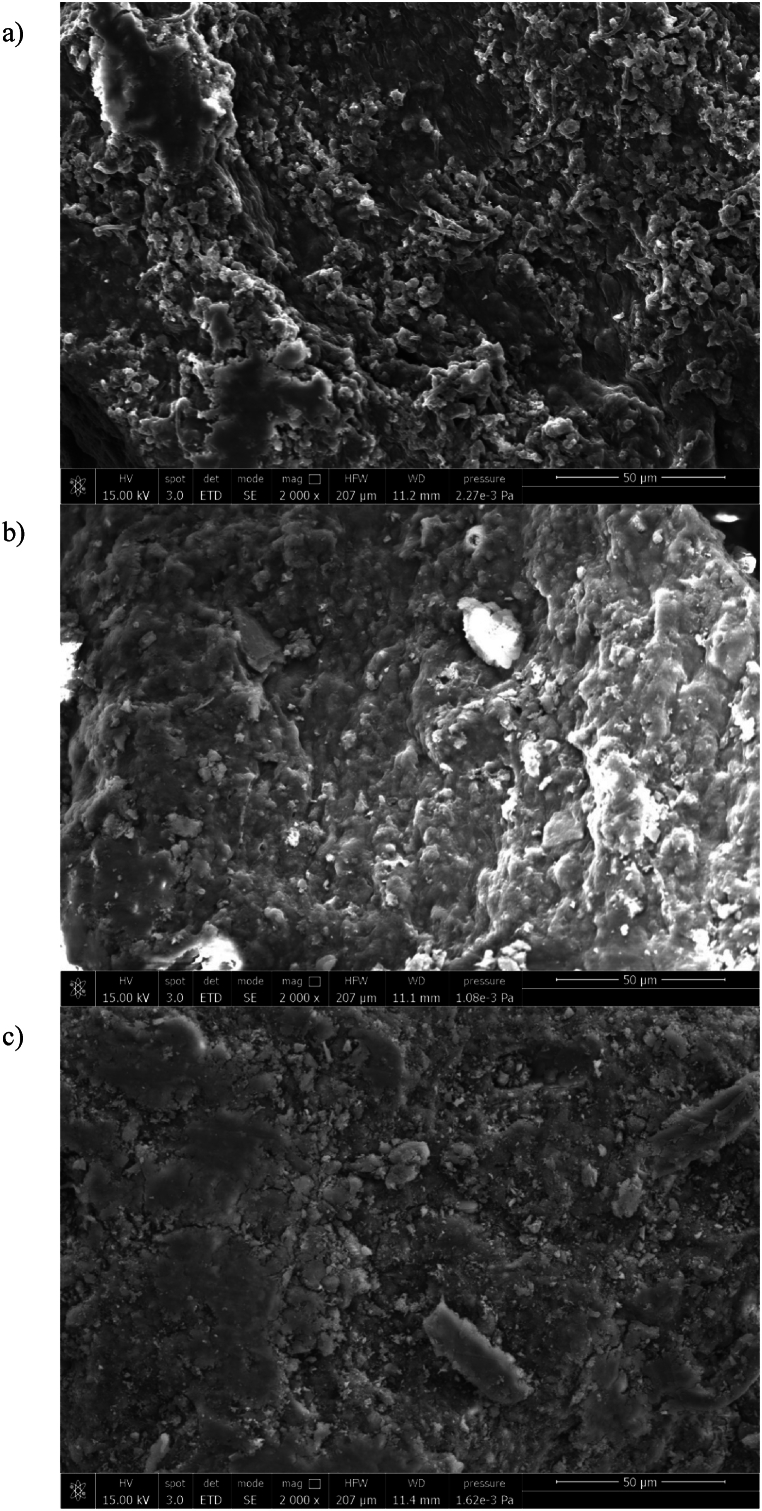
SEM images of control (no coagulant addition) (a), PAC 20 (20 mg Al/g TS of poly aluminum chloride) (b) and AS 20 (20 mg Al/g TS of aluminum sulfate) (c) samples.

Fig. 6 showed SEM images collected at 2000× of magnitude, where the control sample (Fig. 6a) was compared with the samples with a concentration of 20 mg Al/g TS of PAC (Fig. 6b) and AS (Fig. 6c), in order to highlight the differences after the addition of coagulant in WAS. The addition of PAC or AS changed the morphology of WAS; however, this effect was already appreciable with an addition of coagulant equal to 5 mg Al/g TS.

As can be seen, the control sample showed a fibrous structure. With the addition of PAC, the structure appeared compact, suggesting a breakdown of the fibers which gave the sludge a more homogeneous aspect.

The same effect can be observed after the addition of AS, where the fibrous structure disappeared; however, compared to PAC, the sludge structure seemed porous. This result demonstrated that PAC and AS exhibited a different coagulation mechanism, which also affected the morphology of sample [25].

### 3.4 Further insights and potential implications

The results obtained showed that PAC and AS reduced the potential production of methane in anaerobic digestion reactor. These findings can be valuable for wastewater treatment in the near future, in order not to hinder energy recovery, given the growing global demand worldwide [48].

As discussed above, most of the previous studies in the literature have proven that Al and Fe-based coagulants usually inhibit the maximum methane yield. On the contrary, Zhan et al. [20] revealed that FC can improve the methane production up to 197.2%. However, it should be noted that the maximum methane yield was not achieved at the highest FC dosage. At the same time, Wu et al. [48] demonstrated that the addition of PAC decreased the production of SCFA, as also shown by Chen et al. [24], but increased the production of biohydrogen.

On the other hand, the use of coagulants can be a useful strategy to enhance the dewaterability of digested sludge [21], improve sludge volume index (SVI) [25], reduce marine sediment pollution [49] and maximize nutrient recovery [50], which are consumed less during anaerobic digestion process [51].

**Fig. 7:**
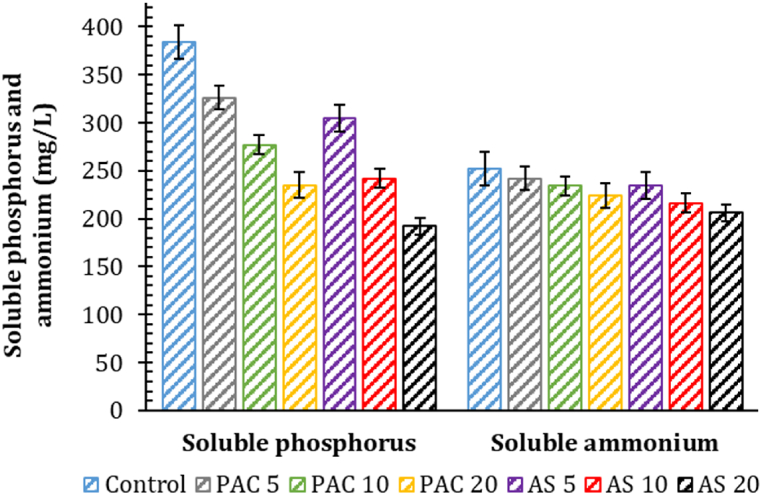
Values of soluble phosphorus and ammonium in waste activated sludge (WAS) after the addition of different poly aluminum chloride (PAC) and aluminum sulfate (AS) levels. The associated numbers in the legend indicate the coagulant level in terms of mg Al/g TS. No coagulants were dosed in the control.

The concentrations of soluble phosphorus and ammonium in WAS after coagulant conditioning were reported in Fig. 7. As shown, both PAC and AS were less efficient for ammonium, probably due to the inability of Al^3+^-based processes in removing positively charged NH_4_^+^-N [52]. Conversely, the increasing level of Al-based coagulant increased the concentration of soluble phosphorus, confirming a great ability to aggregate phosphorus. In this regard, Chen et al. [13] demonstrated that the optimal concentration of PAC to recover phosphorus from primary sludge was equal to 100 mg Al/g TS; however, according to the literature and our study, similar dosages should strongly suppress methane production.

Other nutrients such as potassium generally have a very low content, and their accumulation in WAS may not be significant [5]; for this reason, the analyses were carried out on nutrients of greatest interest in this field [53].

## 4 Conclusions

The aim of this study was to compare the impact of poly aluminum chloride (PAC) and aluminum sulfate (AS) on WAS anaerobic digestion, by feeding replicate serum reactors with different levels of coagulant (0, 5, 10 and 20 mg Al/g TS).

Results revealed that Al-based coagulants inhibited methane production, which decreased as the coagulant addition increased. The inhibition was much severe in AS-conditioned reactors. Analytical analysis, FTIR and SEM investigations revealed that the addition of coagulants affected the initial conditions of the anaerobic reactors, penalizing the solubilization, hydrolysis and acidogenesis phases. Furthermore, the massive formation of H_2_S in AS-conditioned reactors played a key role in the suppression of methane phase. On the other hand, the use of coagulant can promote the accumulation and recovery nutrient in WAS, especially in terms of phosphorus.

Our findings will expand research knowledge in this field and guide stakeholders in the choice of coagulants at full scale plant. Future research should focus on reducing the effect of coagulants on methane production by modifying or testing new types of flocculants.

## Data availability statement

The authors declare that no data associated with our study has been deposited into a publicly available repository. All the data that support the findings of this study are available from the corresponding author upon request.

## CRediT authorship contribution statement

**Francesco Pasciucco:** Writing – original draft, Visualization, Validation, Software, Methodology, Investigation, Formal analysis, Data curation, Conceptualization. **Erika Pasciucco:** Writing – original draft, Visualization, Software, Investigation, Formal analysis, Data curation. **Alessio Castagnoli:** Visualization, Validation. **Renato Iannelli:** Writing – review & editing, Validation, Supervision, Resources, Methodology, Funding acquisition, Conceptualization. **Isabella Pecorini:** Writing – review & editing, Writing – original draft, Supervision, Software, Resources, Project administration, Methodology, Investigation, Funding acquisition, Conceptualization.

## Declaration of competing interest

The authors declare that they have no known competing financial interests or personal relationships that could have appeared to influence the work reported in this paper.
