# Supplementary material for: Comparing the effects of Al-based coagulants in waste activated sludge anaerobic digestion: Methane yield, kinetics and sludge implications
Source: Heliyon. 2024 Apr 7;10(7):e29282. doi: 10.1016/j.heliyon.2024.e29282 (PMC11016704; doi:10.1016/j.heliyon.2024.e29282)
Supplement: Multimedia component 1 [file mmc1.docx]

**Comparing the effects of Al-based coagulants in waste activated sludge anaerobic digestion: biomethane yield, kinetics and sludge implications**

Figure 1: FTIR analysis of control sample.

Figure 2: FTIR analysis of PAC 20 sample.

Figure 3: FTIR analysis of AS 20 sample.
